# Commencement Exercises Buffalo Medical College

**Published:** 1873-03

**Authors:** 


### Editorial.

COMMENCEMENT EXERCISES OF THE MEDICAL DEPARTMENT OF THE UNIVERSITY OF BUFFALO.—The Twenty-Seventh Annual Commencement exercises of this Institution were held at St. James Hall, Tuesday Evening, February, 25th. The Hall was filled by friends of the Graduates and of the Institution. On account of the illness of the Chancellor, Hon. Millard Fillmore, the degrees were conferred by Prof. JAMES P. WHITE, M. D., President of the Council. The following is a list of the Graduates.

William Francis Hutchinson, Minnesota; Eugene Henry Howard, New York; Ambrose Kasson, New York; Joseph Fowler, New York; Clarence Romanzo Seeley, New York; Warren Stewart, New York; John Walter Swanson, New York; Frank Elijah Dewey, New York; Frank Champlin Clarke, New York; John Hugh Van Kleeck, New York; Orrin I. Hall, New York; Henry Alfred Bishop, New York; Orrin Carlton Shaw, New York;

Nathan Cook, New York; Allan Augustus Stevens, Jr., New York; Comfort Edson Peck, New York; Morris Alanson Millard, Pennsylvania; Rollin Thomas Rolph, Pennsylvania; Harry Rocine Nettleton, New York; Emery M. Cheney, New York; John J. Morgan, New York; Derrick V. Crossmire, Pennsylvania; Benjamin A. Fuller, New York; Francis Augustus Burghardt, New York; William Hadley Slacer, New York; Charles Albert Greene, New York; John Charles Bump, New York; James Alfred Barringer, New York; Andrew Jackson, New Jersey; Egbert George, Pennsvlvania; Alfred Tennyson Livingston, A. B., Pennsylvania; Thomas Charles Wilson, New York; DeWitt Clinton Hunter, M. D., New York; Lamarr V. Knapp. Pennsylvania; Charles Henry Johnson, New York; Daniel A. Bailey, New York; Andrew Bresee Stevens, New York; Alonzo Van Allen, Ontario; James A. Williams, Michigan; George Gamble Brush, Pennsylvania.

The address to the Graduating Class was delivered by Prof. J. F. MINER.

The Faculty and Curators recommended the Thesis of ALFRED T. LIVINGSTON, A. B., upon Ten Cases of Excision of Joints, for publication, and we have the pleasure of presenting it to our readers in this issue. The exercises were pleasantly varied by music, and every thing passed off in a pleasant and agreeable manner.

